# Condom use social norms and self-efficacy with different kinds of male partners among Chinese men who have sex with men: results from an online survey

**DOI:** 10.1186/s12889-018-6090-5

**Published:** 2018-10-16

**Authors:** Cheng Wang, Joseph D. Tucker, Chuncheng Liu, Heping Zheng, Weiming Tang, Li Ling

**Affiliations:** 1 Dermatology Hospital, Southern Medical University, Guangzhou, China; 2 Guangdong Center for Skin Diseases and STI Control, Guangzhou, China; 3 University of North Carolina at Chapel Hill Project-China, Guangzhou, China; 4 SESH study group of University of North Carolina at Chapel Hill, Guangzhou, China; 5 School of Medicine of University of North Carolina at Chapel Hill, Chapel Hill, USA; 6 Faculty of Medical Statistics and Epidemiology, School of Public Health, Sun Yat-sen University, Guangzhou, China

**Keywords:** Social norms, Self-efficacy, consistent condom use, men who have sex with men, regular partners, casual partners

## Abstract

**Background:**

Social norms and self-efficacy play important roles in promoting consistent condom use among men who have sex with men (MSM). Few studies have investigated the association between social norms, self-efficacy and consistent condom use with different kinds of male partners among MSM. We conducted an online survey of MSM to evaluate this in China.

**Methods:**

A cross-sectional online survey was conducted in 2015. Participants completed a validated questionnaire covering socio-demographic information, consistent condom use, condom use social norms and self-efficacy. Eligible participants were 16 or older, born biologically as a male, engaged in anal sex with a man at least once during their lifetime, engaged in condomless anal or vaginal sex in the last three months. In this study, we further restricted to people who had sex with male partners in the last three months. Participants were classified into three groups: engaged in sex only with regular partners, engaged in sex only with casual partners and engaged in sex with both regular partners and casual partners.

**Results:**

Participants were recruited from 32 provinces in China. Among 1057 participants, 451(42.7%), 217(20.5%), and 389(36.8%) engaged in sex with regular partners only, casual partners only and both types in the last three months, respectively. Men engaged in sex only with regular partners in the last three months had a higher condom use self-efficacy than with other two types of partners (*P* < 0.01). Both social norms (regular partners: adjusted OR:1.59, 95% CI: 0.97–2.60; casual partners: adjusted OR: 1.58, 95% CI: 1.19–2.09; both types: adjusted OR: 1.48, 95% CI: 1.13–1.95) and self-efficacy (regular partners: adjusted OR: 2.88, 95% CI: 1.59–5.22; casual partners: adjusted OR: 2.35, 95% CI: 1.69–3.26; both types: adjusted OR: 2.45, 95% CI: 1.81–3.32) were positively associated with consistent condom use. No interaction effect was detected between condom social norms and self-efficacy on consistent condom use among Chinese MSM (*p* > 0.05).

**Conclusions:**

Both social norms and self-efficacy were positively correlated with consistent condom use with any types of partners among Chinese MSM. Tailored interventions that aimed to improve social norms and self-efficacy has the potential to improve overall condom use among Chinese MSM.

**Trial registration:**

ClinicalTrials.gov: NCT02516930. August 6, 2015.

**Electronic supplementary material:**

The online version of this article (10.1186/s12889-018-6090-5) contains supplementary material, which is available to authorized users.

## Background

Promoting consistent condom use is an important strategy for HIV/STIs control among men who have sex with men (MSM) [1]. However, studies in China showed that the rate of consistent condom use remains and persists low among Chinese MSM [2–4], even though extensive efforts on promoting consistent condom use had been made (e.g., the 100% Condon Use Program, condom promotion in venues, and peer education) [5, 6]. For example, a national wide serial cross-sectional study showed that only 48.8% of 42,680 MSM had consistently used condoms with men in the last six months in 2013 [7]. Previous studies have documented several reasons for the limited condom use among Chinese MSM, which including but not limited to low HIV risk perception, and low condom use social norms and self-efficacy [8, 9]. Condom use social norms are interpreted as the perception of society’s approval of condom use behavior [10]. Condom use self-efficacy is conceptualized as a person’s confidence in their ability to use condoms [11]. Self-efficacy and social norms are two social cognitive factors that are theorized to play a key role in determining behavior and behavior change [12]. Altering condom use social norms and self-efficacy are considered to be two vital methods for promoting condom use [8, 9].

Quite a few studies have explored the association between consistent condom use, social norms and self-efficacy among MSM [12–16]. These studies indicated that MSM with higher condom use social norms or higher condom use self-efficacy were less likely to engage in condomless sex [13–15]. There have been several studies examining the association among different sexual partner types as well [12, 16]. A study conducted in the United States found that, condom use self-efficacy was associated with decreased rates of unprotected anal intercourse with casual partners [12]. Another study of young gay-identified men in Amsterdam found that intention to use condom was the best predictor of consistent condom use with regular partners, while the best predictor for condom use with casual partners was perceived behavioral control defining as the evaluation of one’s abilities and opportunites to engage in protected anal sex [16]. However, studies on examining the association between social norms, self-efficacy and consistent condom use with different kinds of male partners among Chinese MSM are limited. Given higher levels of social stigma and discrimination [17], and the different likelihood of engaging in condomless intercourse with regular and casual partners among Chinese MSM [18], it is critical to explore the association to support a design of tailored intervention program for Chinese MSM .

Therefore, our study aims to evaluate the patterns of social norms and self-efficacy regarding condom use among Chinese MSM, and their associations with consistent condom use with different kinds of partners.

## Methods

### Study design and participants

This study was the baseline of a randomized control trial which aimed to evaluate the potential effect of crowdsourcing intervention in promoting condom use among Chinese MSM [19]. Crowdsourcing allows a group to solve a problem, then shares the solution widely with the public [20, 21]. For the baseline survey, we conducted a cross-sectional online survey of MSM in China from November 2nd to 7th 2015 (see Additional file 1 for the study questionnaire). Study recruitment was conducted through Danlan.org (the largest online gay portal in China,) and its associated gay mobile dating app (Blued) as well as Weibo (a microblogging platform) and WeChat (a messaging app). A banner advertisement was put on the platforms mentioned above, and participants entered the survey by clicking on the banner, which directed them to a survey website hosted on Qualtrics (Provo, Utah).

All participants who clicked the link for the survey were screened for eligibility. Inclusion criteria included: born biologically as a male, engaged in anal sex with a man at least once during their lifetime, engaged in condomless anal or vaginal sex in the last three months, and at least 16 years of age. In this study, we further restricted to people who engaged in sex with male partners in the last three months.

### Measures

The participants were further classified into three groups according to their reporting sexual partner types in the last three months: engaged in sex only with regular partners, engaged in sex only with casual partners and engaged in sex with both regular partners and casual partners. In our study, the regular partner was defined as main partners to whom the participant feels committed, such as a spouse, lover or boyfriend [22]. A casual partner was defined as any sexual partner that the participant did not consider to be his regular partner [23].

#### Socio-demographic and consistent condom use

Socio-demographic information included: age in completed years (continuous), education (highest grade completed), marital status (not married/married), student (yes/no), annual income and sexual orientation (Gay/bisexual). Participants who reported using condoms every time during sex with male partners in the last three months were considered to be consistent condom users, and those who reported using condoms most of the time, sometimes, and never were considered as inconsistent condom users. The condom use variables were dichotomized as consistent versus inconsistent for the current analysis.

#### Condom use social norms

Condom use social norms were measured using a 6 item subscale from the original scale developed by Dana D. DeHart and John C. Birkimer [24], which intended to measure participants’ perception of their friends’ attitudes towards condom use and safe sex. For example, one item is “If I had sex and told my friends that I did not use a condom, they would be angry or disappointed.” Responses were rated on a 5-point Likert scale: strongly agree (5), agree (4), neutral (3), disagree (2), strongly disagree (1). The subscale has been widely validated and used among college students [24] and MSM [25, 26]. The detailed information for the sex items was listed in the Supplementary Table [see Additional file 1: Table S1]. In the present study, the internal consistency (Cronbach’s alpha) for the six items was 0.770 [27]. The overall score was calculated for each participant, which a higher score indicated the higher self-reported strength of social norm for condom use.

#### Condom use self-efficacy

This is a 7-item scale that measures self-efficacy on using condoms in multiple situations and settings [28]. Participants again responded to statements on a 5-point Likert format ranging from 1 strongly disagree to 5 strongly agree. These seven items were selected from the Condom Use Self-Efficacy Scale developed by Brafford and Beck’s (1991) [28] to measure participants’ confidence in being able to use a condom and in being able to communicate with a partner about condom use. For example, one item is “I feel confident that I could refuse to have sex with a partner who did not want me to use a condom.” The Condom Use Self-Efficacy Scale has been validated and applied among MSM [29, 30] and other populations [31]. In the current study, the Cronbach’s alpha for the scale was 0.823 [27]. We also calculated the overall score for each participant, with higher scores indicating greater comdom use self-efficacy. The seven items were listed in the Supplementary Table as well [see Additional file 2: Table S1].

### Statistical analysis

Data analysis was performed using SPSS version 17.0. Descriptive statistics were used to describe socio-demographics, consistent condom use, condom social norms and self-efficacy.

Univariate and multivariable logistic regressions were used to determine the association between condom use social norms, condom use self-efficacy and consistent condom use with different kinds of partners, while demographic characteristics, including age, marital status, and income, were adjusted in the multivariable logistic regression models.

Logistic regression and maximum likelihood parameter estimation methods were used to analyze the multiplicative effect and additive effect between condom social norms and self-efficacy on the consistent condom use among all participants, respectively.

## Results

A total of 1597 participants meeting inclusion criteria and providing informed consent were recruited from 269 cities in 32 out of 34 provinces of China. Of those participants, 1189 completed the survey, with a completion rate of 74.5%. One hundred and thirty-two participants were further excluded, as they did not report the type of sexual partner they had sex with during the last three months. Overall, 1057 participants who have male partners were included in this study.

### Study participants

Of the 1057 participants, 451 (42.7%), 217 (20.5%), and 389 (36.8%) engaged in sex with regular partners only, casual partners only and both types in the last three months, respectively. The majority were less than 25 years old (63.9%, 673/1054), not students (63.6%, 672/1057), never married (83.3%, 881/1057), had a college degree (63.3%, 670/1057), and had an annual income less than $5000 USD (53.8%, 569/1057). Nearly three-fourth participants self-identified as gay (71.1%, 751/1057), of whom 82.8% (622/751) engaged in condomless sex in the last three month. Almost one-third self-identified as bisexual (28.9%, 306/1057), and 76.5% (234/306) of those engaged in condomless sex in the last three month. One-fourth (19.8%, 43/174) of casual partners were paid (with money or gifts) to have sex. The rate of consistent condom use with any type of male partners in the last three months was only 15.1% (151/1001). 96.2% (1017/1057) of participants engaged in condomless anal intercourse in the last three month. Men engaged in sex only with casual partners (19.8%, 36/182) had a higher rate of consistent condom use than only with regular partners (13.5%, 61/451) or both types (13.2%, 40/264) (Table 1).

**Table 1:** Socio-demographic characteristics and condom use among high-risk MSM with different kinds of partners in China, 2015 (*N* = 1057)

		Regular partner		Casual partner		Both types		Total^a^	
		No.	%	No.	%	No.	%	No.	%
Age(yrs)	16–25	292	64.9	147	68.1	234	60.3	673	63.9
	26–35	131	29.1	52	24.1	115	29.6	298	28.3
	36–45	23	5.1	10	4.6	32	8.2	65	6.2
	> 45	4	0.8	7	3.2	7	1.8	18	1.7
Education	High school or below	142	31.5	80	36.9	116	29.8	338	32
	College	287	63.6	127	58.5	256	65.8	670	63.3
	Graduate education	22	4.9	10	4.6	17	4.4	49	4.6
Marital status	Not married	381	84.5	187	86.2	313	80.5	881	83.3
	Married	70	15.5	30	13.8	76	19.6	176	16.6
Student	Yes	174	38.6	98	45.2	113	29	385	36.4
	No	277	61.4	119	54.8	276	71	672	63.6
Annualincome (USD)	5000 or less	252	55.9	137	63.2	180	46.2	569	53.8
	5001–15,000	166	36.8	70	32.3	182	46.8	418	39.5
	> 15,000	33	7.3	10	4.6	27	6.9	70	6.6
Sexual orientation	Gay	329	72.9	154	71	268	68.9	751	71.1
	Bisexual	122	27	63	29	121	31.1	306	28.9
Paid to have sex in the last 12 month	Yes	41	9.1	43	19.8	53	13.6	137	13.0
	No	410	90.9	174	80.2	336	86.4	920	87.0
Consistently condom use with male partners	Yes	61	13.5	36	19.8	40 ^b^	13.2	151	15.1
	No	377	86.1	146	80.2	264	86.8	850	84.9

^a^the whole population

^b.^These participants engaged in condomless sex with female partners in the last three months

### Condom use social norms and self-efficacy characteristics

The median (IQR) score of condom use social norms for men engaged in sex with regular partners, casual partners and both regular and casual partners were 23 (22.2–22.9), 22 (21.6–22.7) and 23 (22.3–23.1), respectively. However, there is no statistical difference in the median score among those three types (*P* > 0.05).

The median (IQR) score of condom use self-efficacy for men engaged in sex with regular partners, casual partners and both regular and casual partners were 29 (27.9–28.8), 26 (26.1–27.4) and 28 (27.4–28.4), respectively. Men engaged in sex only with regular partners had a higher median score of condom use self-efficacy than with other two different types of partners (*P* < 0.01).

### Multivariable analysis

For men only engaged in sex with regular partners, both social norms (adjusted OR: 1.59, 95% CI: 0.97–2.60) and self-efficacy (adjusted OR: 2.88, 95% CI: 1.59–5.22) were positively associated with consistent condom use. For men only engaged in sex with casual partners, participants with higher social norms (adjusted OR: 1.58, 95% CI: 1.19–2.09) and higher self-efficacy (adjusted OR: 2.35, 95% CI: 1.69–3.26) were more likely to use a condom consistently. Again, for men engaged in sex with both regular and casual partners, both social norms (adjusted OR:1.48, 95% CI: 1.13–1.95) and self-efficacy (adjusted OR: 2.45, 95% CI: 1.81–3.32) were significantly correlated with consistent condom use. No multiplicative (OR: 0.99, 95% CI: 0.98–1.01) and additive effect were detected between social norms and self-efficacy on consistent condom use among MSM (*p* > 0.05) (Tables 2, 3 and 4).

**Table 2:** Factors associated with consistent condom use among high-risk MSM with different sex partner types in China, 2015 (*N* = 1057)

Variables	Both types^a^		Regular^a^		Casual^a^	
	Crude model	Adjusted model	Crude model	Adjusted model ^b^	Crude model	Adjusted model^b^
	OR (95% CI)	OR (95% CI)	OR (95% CI)	OR (95% CI)	OR (95% CI)	OR (95% CI)
Student						
Yes	Ref.	Ref.	Ref.	Ref.	Ref.	Ref.
No	1.04(0.71–1.51)	1.06(0.66–1.71)	0.93(0.63–1.38)	0.82(0.5–1.34)	1.23(0.83–1.84)	1.14(0.7–1.87)
Sexual orientation						
Gay	0.53(0.23–1.26)	0.52(0.22–1.24)	0.59(0.24–1.41)	0.60(0.25–1.45)	1.47(0.46–4.71)	1.46(0.45–4.73)
Bisexual	0.84(0.35–2.06)	0.86(0.35–2.1)	0.66(0.26–1.67)	0.68(0.27–1.72)	1.64(0.5–5.43)	1.65(0.5–5.49)
Unsure/other	Ref.	Ref.	Ref.	Ref.	Ref.	Ref.
Education						
High school or below	1.1(0.41–2.96)	1.09(0.4–2.98)	0.81(0.31–2.08)	0.83(0.31–2.2)	0.55(0.22–1.37)	0.58(0.23–1.47)
Some college	1.98(0.74–5.27)	1.97(0.73–5.31)	1.44(0.57–3.68)	1.48(0.57–3.84)	1.02(0.41–2.52)	1.04(0.41–2.58)
College/Bachelors	1.15(0.43–3.07)	1.11(0.41–2.98)	0.83(0.33–2.11)	0.84(0.32–2.17)	1.04(0.43–2.54)	1.05(0.43–2.59)
Masters or PhD	Ref.	Ref.	Ref.	Ref.	Ref.	Ref.
Social norms	1.61(0.98–2.64)*	1.59(0.97-2.60)*	1.59(1.20-2.09)*	1.58(1.19-2.09)*	1.49(1.14-1.95)*	1.48(1.13-1.95)*
Self-efficacy	2.92(1.62–5.25)*	2.88(1.59-5.22)*	2.36(1.70-3.26)*	2.35(1.69-3.26)*	2.50(1.85-3.37)*	2.45(1.81-3.32)*

*p < 0.01

^a^Adjusted model was adjusted for age(continuous),income and marital status

**Table 3:** Multiplicative effect between social norms and self-efficacy on consistent condom use among Chinese high-risk MSM, 2015 (*N* = 1057)

Variables	B	SE B	Wald	OR (95% CI)
Social norms	0.15	0.16	0.88	1.17(0.84–1.60)
Self-efficacy	0.24	0.13	3.72	1.27(0.99–1.63)
Social norms × Self-efficacy	−0.01	0.01	0.95	0.99(0.98–1.01)

**Table 4:** Additive effect on consistent condom use between social norms and self-efficacy among Chinese high-risk MSM, 2015 (*N* = 1057)

Variables	B	*SE* B	Wald	95% CI	
				lower	upper
Social norms	0.019	0.02	0.35	0.01	0.02
Self-efficacy	−0.0004	0.02	0	−0.04	0.0009
Social norms +Self-efficacy	0	0.0007	0	0	0.0001

## Discussion

Knowing the situation of condom use social norms and self-efficacy among Chinese MSM are critical for designing tailored interventions to initiate and sustain condom use. Our study extended the existing literature by evaluating the pattern of self-efficacy and social norms among Chinese MSM, assessing the association with different types of partners among Chinese MSM, and exploring the interaction between social norms and self-efficacy on consistent condom use. Our findings indicated that condom use social norms and self-efficacy were positively associated with consistent condom use with all the three types of partners.

Our study observed that for men who have higher condom use social norms are more likely to use condoms consistently with either regular partners or casual partners. These findings are consistent with the results of previous studies that focused on exploring the association with all partners together [32–34]. For example, a study conducted among Chineses MSM found positive correlations between social norms and practices of condom use [33]. As indicated in the previous studies [13, 34], one of the reasons for this phenomenon is that men who perceive stronger social norms for condom use were more likely to hold stronger beliefs in their ability to use condoms, and had stronger intentions to practice safer sex. Additionally, we also found that a large minority (7.7 to 29.5%) of participants were not able to endorse every item of social norms. An effective intervention strategy for promoting condom use typically was determined by three components: (a) attitudes, (b) social norms, and (c) self-efficacy [35]. That is, the intervention strategy could make participants feel positively about always use a condom, increase participants’ perception of society’s approval of condom use, and believe that one could use a condom consistently [35]. Our results echoed this finding of social norms being an important part of an effective intervention, and encourage an inclusive of peer intervention to strengthen social norm regarding condom use [36].

Similarly, our results pointed out that condom use social self-efficacy played a positive role in facilitating consistent condom use with any types of partners. These findings were also observed in previous studies aiming to explore the association with all partners together [33, 37, 38]. For example, two studies conducted in the United States [37, 38] and one study in China [33] among MSM suggested that enhancing self-efficacy is an important factor improving condom use behavior. Additionally, our study found that men engaged in sex only with regular partners in the last three months have the highest condom use self-efficacy, specifically for the setting of refusing to have sex with a partner who did not want to use a condom. This phenomenon can be explained by the committed and trustful relationship with regular partners [39]. This highlights a need to make tailored interventions, such as cognitive-behavioral interventions (e.g., interactive counseling) [38], to enhance condom use self-efficacy when engaging in sex with casual partners among MSM.

To know the interaction between social norms and self-efficacy would help us to further understand the mechanism of how social norms and self-efficacy working together on altering condom use. Several studies reported that social norms may have an indirect effect on condom use via self-efficacy [40, 41]. One study conducted among American MSM pointed out that the effect of social norms on unsafe sex was diminished once the effect of self-efficacy was taken into account [12]. However, we did not identify the interaction in the current study, neither for any kind of partner types. One potential reason for this phenomenon is the participants in our study are extremely skewed, as all of them were either engaged in condomless sex with male or female in the last three months, which limited the power to test this interaction (the power for the multiplicative effect and additive effect in this study is 0.032 and 0.025, respectively). This may explain why only 15% of participants in this study consistently used a condom with male partners in the last three months.

Our study also showed that men engaged in sex only with casual partners have a higher rate of consistent condom use than with the other two types of partners. This finding is inconsistent with a study conducted in India [22] but supported by other studies conducted in China, which showed that condomless sex among MSM was more likely to occur when regular partners were involved [42–44]. The potential reasons for this include that MSM have a higher awareness of self-protection when having sex with casual partners [45], and using a condom with regular partners would be considered as an insult to the partner [39]. Even like this, previous studies indicated that a large proportion of Chinese MSM persistently engaged in condomless sex with casual partners [46, 47]. Considering multi-sexual partnerships are common, and HIV prevalence is high [48, 49], there is a high likelihood that the HIV burden among Chinese MSM will further worsen. To avert this trend, intervention package that including social norms and self-efficacy promotion strategies are further needed.

Our study has several limitations. First, our study was conducted online, and the participants tended to be younger and more educated, which might lead to underestimation of the findings when generalized to the whole MSM population in China [50]. Second, all the data was obtained through self-reported, which may be influenced by social desirability bias. This bias could cause underestimation of the association between social-norms and self-efficacy on consistent condom use. However, a computer-based survey has the potential to reduce this kind of bias. Third, this study was cross-sectional, so relations should be interpreted as associations that might or might not be causal. Fourth, the data we used for this study were baseline data of a randomized control trial that aimed to promote condom use, while all the participants either engaged in condomless sex with male or female in the last three months. Last but not the least, 19.8% of the participants who have casual partners had sexual clients in the last 12 months, and we did not separate clients from other casual partners, while these two groups of partners are potentially different.

## Conclusion

To conclude, altering condom use social norms and self-efficacy are two promising strategies to promote condom use among Chinese MSM. Social norms and self-efficacy are positively associated with consistent condom use with either regular partners or casual partners, further suggesting the importance of structural intervention on promoting condom use based on social norm and self-efficacy theories. Future studies that aimed to understand the mechanism on how social norm and self-efficacy work together on altering condom use are still needed.

## Additional files

Additional file 1: Study questionnaire. (DOCX 86 kb)

Additional file 2: **Table S1.** The list of items of Social norms and Social self-efficacy. (DOCX 36kb)

## Abbreviations

HIV: Human immunodeficiency virus
MSM: Men who have sex with men
USD: US dollars

## References

[1] Musinguzi Geofrey, Bastiaens Hilde, Matovu Joseph K. B., Nuwaha Fred, Mujisha Geoffrey, Kiguli Juliet, Arinaitwe Jim, Van Geertruyden Jean-Pierre, Wanyenze Rhoda K. (2015). Barriers to Condom Use among High Risk Men Who Have Sex with Men in Uganda: A Qualitative Study. PLOS ONE.

[2] Guo Y, Li X, Song Y, Liu Y (2012). Bisexual behavior among Chinese young migrant men who have sex with men: implications for HIV prevention and intervention. AIDS Care.

[3] Wei C, Guadamuz TE, Stall R, Wong FY (2009). STD prevalence, risky sexual behaviors, and sex with women in a national sample of Chinese men who have sex with men. Am J Public Health.

[4] Kong TS, Laidler KJ, Pang H (2012). Relationship type, condom use and HIV/AIDS risks among men who have sex with men in six Chinese cities. AIDS Care.

[5] Tang S, Tang W, Meyers K, Chan P, Chen Z, Tucker JD (2016). HIV epidemiology and responses among men who have sex with men and transgender individuals in China: a scoping review. BMC Infect Dis.

[6] Zhongdan C, Schilling RF, Shanbo W, Caiyan C, Wang Z, Jianguo S (2008). The 100% Condom Use Program: A demonstration in Wuhan, China. Eval Program Plann.

[7] Qin Q, Tang W, Ge L, Li D, Mahapatra T, Wang L, Guo W, Cui Y, Sun J. Changing trend of HIV, Syphilis and Hepatitis C among Men Who Have Sex with Men in China. Sci Rep. 2016;6:31081. 10.1038/srep31081 PMC4989164 27535092

[8] Lau JT, Cai W, Tsui HY, Chen L, Cheng J, Lin C, Gu J, Hao C (2012). Unprotected anal intercourse behavior and intention among male sex workers in Shenzhen serving cross-boundary male clients coming from Hong Kong, China–prevalence and associated factors. AIDS Care.

[9] Cai Y, Lau JT. Multi-dimensional factors associated with unprotected anal intercourse with regular partners among Chinese men who have sex with men in Hong Kong: a respondent-driven sampling survey. BMC infectious diseases. 2014;14:205. 10.1186/1471-2334-14-205 PMC3996134 24735186

[10] Latkin CA, Forman V, Knowlton A, Sherman S (2003). Norms, social networks, and HIV-related risk behaviors among urban disadvantaged drug users. Soc Sci Med.

[11] Thorpe L, Ford K, Fajans P, Wirawan DN (1997). Correlates of condom use among female prostitutes and tourist clients in Bali, Indonesia. AIDS Care.

[12] Semple SJ, Patterson TL, Grant I (2000). Partner type and sexual risk behavior among HIV positive gay and bisexual men: social cognitive correlates. AIDS Educ Prev.

[13] Miner MH, Peterson JL, Welles SL, Jacoby SM, Simon Rosser B (2009). How do social norms impact HIV sexual risk behavior in HIV-positive men who have sex with men? Multiple mediator effects. J Health Psychol.

[14] Wagner GJ, Hoover M, Green H, Tohme J, Mokhbat J (2015). Social, relational and network determinants of unprotected anal sex and HIV testing among men who have sex with men in Beirut, Lebanon. Int J Sexual Health.

[15] Sohn A, Cho B (2012). Knowledge, attitudes, and sexual behaviors in HIV/AIDS and predictors affecting condom use among men who have sex with men in South Korea. Osong Public Health and Research Perspectives.

[16] Janssen M, De Wit J, Stroebe W, Griensven F (2000). Educational Status and Risk of HIV in Young Gay Men. J Health Psychol.

[17] Wei C, Cheung DH, Yan H, Li J, Shi L-e, Raymond HF (2016). The Impact of Homophobia and HIV Stigma on HIV Testing Uptake among Chinese Men Who Have Sex with Men: A Mediation Analysis. Journal of acquired immune deficiency syndromes (1999).

[18] Zhou N, Bauermeister J, Guo W, Yu M, Yang J, Zheng M, Guo Y, Gong H, Gao Y, Jiang G (2018). Condomless Anal Intercourse by Partner Type Among Chinese Men Who Have Sex With Men in Tianjin. AIDS Educ Prevent.

[19] Tang Weiming, Mao Jessica, Liu Chuncheng, Mollan Katie, Li Haochu, Wong Terrence, Zhang Ye, Tang Songyuan, Hudgens Michael, Qin Yilu, Ma Baoli, Liao Meizhen, Yang Bin, Ma Wei, Kang Dianmin, Wei Chongyi, Tucker Joseph D (2016). Crowdsourcing health communication about condom use in men who have sex with men in China: a randomised controlled trial. The Lancet.

[20] Tang W, Han L, Best J, Zhang Y, Mollan K, Kim J, Liu F, Hudgens M, Bayus B, Terris-Prestholt F (2016). Crowdsourcing HIV Test Promotion Videos: A Noninferiority Randomized Controlled Trial in China. Clin Infect Dis.

[21] Tang W, Wei C, Cao B, Wu D, Li KT, Lu H, Ma W, Kang D, Li H, Liao M (2018). Crowdsourcing to expand HIV testing among men who have sex with men in China: A closed cohort stepped wedge cluster randomized controlled trial. PLoS Med.

[22] Ramanathan S, Chakrapani V, Ramakrishnan L, Goswami P, Yadav D, Subramanian T, George B, Paranjape R (2013). Consistent condom use with regular, paying, and casual male partners and associated factors among men who have sex with men in Tamil Nadu, India: findings from an assessment of a large-scale HIV prevention program. BMC Public Health.

[23] Lociciro S, Jeannin A, Dubois-Arber F (2013). Men having sex with men serosorting with casual partners: who, how much, and what risk factors in Switzerland, 2007-2009. BMC Public Health.

[24] DeHart DD, Birkimer JC (1997). Trying to practice safer sex: Development of the sexual risks scale. J Sex Res.

[25] Coleman CL, Jemmott L, Jemmott JB, Strumpf N, Ratcliffe S (2009). Development of an HIV risk reduction intervention for older seropositive African American men. AIDS Patient Care STDs.

[26] Frye V, Nandi V, Egan JE, Cerda M, Rundle A, Quinn JW, Sheehan D, Ompad DC, Van Tieu H, Greene E (2017). Associations among neighborhood characteristics and sexual risk behavior among black and white MSM living in a major urban area. AIDS Behav.

[27] Li H, Xue L, Tucker JD, Wei C, Durvasula M, Hu W, Kang D, Liao M, Tang W, Ma W. Condom use peer norms and self-efficacy as mediators between community engagement and condom use among Chinese men who have sex with men. BMC Public Health. 2017;17(1):641. 10.1186/s12889-017-4662-4 PMC5545844 28784172

[28] Brafford LJ, Beck KH (1991). Development and validation of a condom self-efficacy scale for college students. J Am Coll Heal.

[29] Reisner SL, Hughto JMW, Pardee DJ, Kuhns L, Garofalo R, Mimiaga MJ (2016). LifeSkills for Men (LS4M): Pilot Evaluation of a Gender-Affirmative HIV and STI Prevention Intervention for Young Adult Transgender Men Who Have Sex with Men. J Urban Health.

[30] Frye V, Nandi V, Egan J, Cerda M, Greene E, Van Tieu H, Ompad DC, Hoover DR, Lucy D, Baez E (2015). Sexual Orientation- and Race-Based Discrimination and Sexual HIV Risk Behavior Among Urban MSM. AIDS Behav.

[31] Theall KP, Sterk CE, Elifson KW, Kidder D (2003). Factors Associated with Positive HIV Serostatus among Women Who Use Drugs: Continued Evidence for Expanding Factors of Influence. Public Health Rep.

[32] Lau J, Lin C, Hao C, Wu X, Gu J (2011). Public health challenges of the emerging HIV epidemic among men who have sex with men in China. Public Health.

[33] Hu Y, Lu H, Raymond HF, Sun Y, Sun J, Jia Y, He X, Fan S, Xiao Y, McFarland W (2014). Measures of condom and safer sex social norms and stigma towards HIV/AIDS among Beijing MSM. AIDS Behav.

[34] Amirkhanian YA, Kelly JA, Kirsanova AV, DiFranceisco W, Khoursine RA, Semenov AV, Rozmanova VN (2006). HIV risk behaviour patterns, predictors, and sexually transmitted disease prevalence in the social networks of young men who have sex with men in St Petersburg, Russia. Int J STD AIDS.

[35] Fishbein M, Hennessy M, Kamb M, Bolan GA, Hoxworth T, Iatesta M, Rhodes F, Zenilman JM (2001). Using intervention theory to model factors influencing behavior change: Project RESPECT. Eval Health Prof.

[36] Liu H, Liu H, Cai Y, Rhodes AG, Hong F (2009). Money boys, HIV risks, and the associations between norms and safer sex: a respondent-driven sampling study in Shenzhen, China. AIDS Behav.

[37] Hart TA, Heimberg RG (2005). Social anxiety as a risk factor for unprotected intercourse among gay and bisexual male youth. AIDS Behav.

[38] Safren SA, Blashill AJ, Lee JS, O'Cleirigh C, Tomassili J, Biello KB, Mimiaga MJ, Mayer KH (2018). Condom-use self-efficacy as a mediator between syndemics and condomless sex in men who have sex with men (MSM). Health Psychol.

[39] Chamratrithirong A, Kaiser P (2012). The dynamics of condom use with regular and casual partners: analysis of the 2006 national sexual behavior survey of Thailand. PLoS One.

[40] Newcomb ME, Mustanski B (2014). Cognitive influences on sexual risk and risk appraisals in men who have sex with men. Health Psychol.

[41] Jemmott Iii JB, Jemmott LS, O’Leary A, Icard LD, Rutledge SE, Stevens R, Hsu J, Stephens AJ (2015). On the Efficacy and Mediation of a One-on-One HIV Risk-Reduction Intervention for African American Men Who Have Sex with Men: A Randomized Controlled Trial. AIDS Behav.

[42] Chow EPF, Wilson DP, Zhang L (2012). Patterns of Condom Use Among Men Who Have Sex with Men in China: A Systematic Review and Meta-Analysis. AIDS Behav.

[43] Elford J, Bolding G, Maguire M, Sherr L (1999). Sexual risk behaviour among gay men in a relationship. AIDS.

[44] Lau JTF, Cai W, Tsui HY, Cheng J, Chen L, Choi KC, Lin C (2013). Prevalence and Correlates of Unprotected Anal Intercourse Among Hong Kong Men Who Have Sex with Men Traveling to Shenzhen, China. AIDS Behav.

[45] Kumar GA, Dandona R, Poluru R, Chandran SA, Alary M, Dandona L (2014). Patterns of condom use by men who have sex with men before and after the Avahan intervention in Andhra Pradesh state of India. BMC Public Health.

[46] Zhang L, Chow EPF, Wilson DP (2012). Distributions and trends in sexual behaviors and HIV incidence among men who have sex with men in China. BMC Public Health.

[47] Hao C, Lau JTF, Zhao X, Yang H, Huan X, Yan H, Gu J (2014). Associations Between Perceived Characteristics of the Peer Social Network Involving Significant Others and Risk of HIV Transmission Among Men Who Have Sex with Men in China. AIDS Behav.

[48] Chow EP, Jing J, Feng Y, Min D, Zhang J, Wilson DP, Zhang X, Zhang L (2013). Pattern of HIV testing and multiple sexual partnerships among men who have sex with men in China. BMC Infect Dis.

[49] Mitchell JW, Petroll AE (2013). Factors Associated with Men in HIV-Negative Gay Couples Who Practiced UAI Within and Outside of Their Relationship. AIDS Behav.

[50] Wang Cheng, Mollan Katie R, Hudgens Michael G, Tucker Joseph D, Zheng Heping, Tang Weiming, Ling Li (2017). Generalisability of an online randomised controlled trial: an empirical analysis. Journal of Epidemiology and Community Health.

