# Supplementary material for: Condom use social norms and self-efficacy with different kinds of male partners among Chinese men who have sex with men: results from an online survey
Source: BMC Public Health. 2018 Oct 16;18:1175. doi: 10.1186/s12889-018-6090-5 (PMC6192108; doi:10.1186/s12889-018-6090-5)
Supplement: Supplementary file 1 — Study questionnaire. (DOCX 86 kb) [file 12889_2018_6090_MOESM1_ESM.docx]

**Additional file 1 The list of included items of Social norms and Social self-efficacy**

| \| **Social norms** \| \| --- \| \| 1. If I had sex and told my friends that I did not use a condom, they would be angry or disappointed \| \| 2. Friends talk a lot about" safer" sex \| \| 3. My friends and I encourage each other before dates to practice "safer" sex \| \| 4.If I thought that one of my friends had sex on a date, I would ask them if they used a condom \| \| 5. If a friend knew that I might have sex on a date, he/she would ask me if I was carrying a condom \| \| 6. When I think one of my friends might have sex on a date, I would ask him/her if he/she was  carrying a condom \| \| **Self-efficacy** \| \| 1. If I might have sex on a date and I do not have a condom, I would make an effort to get one \| \| 2. I would feel comfortable discussing condom use with a potential partner before we engaged in sex \| \| 3. I would feel comfortable letting a primary partner know that I want to have sex with a condom \| \| 4. I would feel comfortable letting a casual partner know that I want to have sex with a condom \| \| 5. I feel confident that I could refuse to have sex with a partner who did not want you to use a  condom \| \| 6. I feel confident in my ability to incorporate putting a condom on myself or my partner into  foreplay \| \| 7. I feel confident that I could use a condom with a partner without "breaking the mood \| | |
| --- | --- | --- | --- | --- | --- | --- | --- | --- | --- | --- | --- | --- | --- | --- | --- | --- |
|  |  |
|  |  |
|  |  |
|  |  |
|  |  |
|  |  |
|  |  |
|  |  |
|  |  |
|  |  |
|  |  |
|  |  |
|  |  |
|  |  |
