# Supplementary material for: Condom use social norms and self-efficacy with different kinds of male partners among Chinese men who have sex with men: results from an online survey
Source: BMC Public Health. 2018 Oct 16;18:1175. doi: 10.1186/s12889-018-6090-5 (PMC6192108; doi:10.1186/s12889-018-6090-5)
Supplement: Supplementary file 2 — Table S1. The list of items of Social norms and Social self-efficacy. (DOCX 36kb) [file 12889_2018_6090_MOESM2_ESM.docx]

**Men’s Heath Study (Final)**

http://unc.az1.qualtrics.com/jfe/form/SV_9tnFOerTSQi5VJj

About this Study:

You are being asked to take part in a researchstudy that will help us better understand sexual behavior and condom use among men in China. Your participation in this project will allow us to develop better interventions to promote condom use and toimprove sexual health among men across China.

What’s Involved?

If you participate in this study, you will be asked to complete an online questionnaire and a subset of participants will be asked watch a one minute video. A subset of participants will also be asked to complete up to two additional follow-up questionnaires.The questionnaires will ask you to provide sociodemographic information and information about your sexual behaviors. In order to ensure that your privacy is protected, all of your online responses will be encrypted and securely transferred to our data servers.

Upon completion of this study and a 3-week follow up survey, **you will receive 100RMB credit** to your mobile phone. Eligible participants who also complete the follow-up questionnaires can **receive up to 150 RMB credit** to their mobile phone.

If you have any questions about the research or your participation in the study, feel free to contact Chuncheng Liu at (186-1306-7997).

Please make sure the website you are viewed now is start from “http”, not “https”. The later could cause trouble in viewing the video and incomplete of the survey. Plese change your browser and visit: <http://unc.az1.qualtrics.com/jfe/form/SV_9tnFOerTSQi5VJj>if the page is “https” now.

**A.Basic Information (Eligibility Survey) (Q1-5)**

A1. Were you born biologically male or female?

- Male
- Female (Not eligible to take this survey – Skip to End of Survey)

A2. What is your date of birth?

- dd.mm.yyy (*Calendar input*)(Not eligible to take this survey if year is greater than Launch day + 1999 or < 16 y/o – Skip to End of Survey)

A3. In your lifetime have you ever had anal sex with another man?

- Yes
- No (Not eligible to take this survey – Skip to End of Survey)

A4. In the last three months, did you have any anal and /or vaginal sex without a condom with any sex partner?

- Yes
- No (Not eligible to take this survey – Skip to End of Survey)

A5. Will you agree to provide us your Chinese mobile phone number? (Answering this question is required to participate in the survey and to receive your reward for participating. We will not distribute your number to any agency or individual. Thank you for your cooperation.)

- Agree
- Decline (Not eligible to take this survey – Skip to End of Survey)

Which carrier are you using right now?

- China Mobile
- China Unicom
- China Telecom

**Online Consent Form**

**Title of Study:** Men’s Health Study

**IRB study number: 15-1522
Principal Investigator:** Dr. Joseph Tucker
Dr. Joseph D. Tucker, UNC Project-China, Number 2 Lujing Road, Guangzhou, China, [jdtucker@med.unc.edu](mailto:jdtucker@med.unc.edu)

**What are some general things you should know about research studies?** You are being asked to participate in a research study. To join this researchstudy is voluntary. You may for whatever reason refuse to join or withdraw your consent to be in the study at any time, without penalty. Details about this study are discussed below. It is important that you understand this information so that you can make an informed choice about joining this research study.

**What is the purpose of this study?** Innovative approaches to condom promotion campaigns are urgently needed. The current strategy to developing many of these campaigns is to repackage old ideas rather than create new ones. The purpose of this research study is to understand how crowdsourcing can be used to leverage both the high Internet use and willingness to participate in online forums ofyoung MSM (men who have sex with men) to transform the design and implementation of condom promotion campaigns. Crowdsourcing is the process of taking a task traditionally performedby a single individual or organization, and instead outsourcing the taskto a large group to complete in the form of a contest or open call, often enabled by the Internet.

**How many people will take part in this study?** If you decide to participatein this research study, you will be one of approximately 1169individuals recruited across China.

**What will happen if you take part in the study?** Your part in this research study will last approximately 20 minutes. During this study, you will be asked to firstcomplete an online questionnaire,anddepending on your responses,you maybe asked to watch aone minute video afterwards. Upon completion of this initial questionnaire,you will be asked to input your mobile phone number as a means for the research team to prevent duplicate responses, to send reminders, and to distribute rewards for participation. Additionally, some participant will be asked to complete up to two additional follow-up questionnaires after three-week and twelve-week’s times. If you do not respond to the initial follow-up request, you will receive a message reminder. To do this, we will also ask you to provide your QQ number. Thestudy questionnaires will ask you to provide sociodemographic informationas well as details about your sexual health and sexual activity.

**What are the possible benefits from being inthis study?** Research is designed to benefit society by gaining new knowledge. The proposed study will make important contributions to the sexual health literature. The field of condominterventions among young MSM in resource-limited settings is in its infancy. The results from thisstudywill help the research team develop a MSM targeted, community-level intervention that will be fielded and evaluated in the Chinese setting. Your participation will also help design better interventions to promote condom use among MSM in China.

**What are the possible risks or discomforts involved from being in this study?** We will ask participants to provide sensitive information about their sexual partners and practices. Participants may feel embarrassed, anxious, or otherwise distressed by providing information of such a personal nature. Participants may also experience fatigue in response to the proposed evaluations (e.g. from looking at a computer screen). Some participants might fear that refusal to participate in the study might jeopardize their sexual orientation identity – especially if the participant has not come“out” to him or herself and/or the community). Other participants may fear that the research staff might “out” them or discuss theirprivate details with other (MSM and non-MSM) members in their community. While the risk is minimal, there is still the possibility for breaches of confidentiality.

**How will your privacy be protected?**All data are directly entered into computers as participants complete the questionnaires. Programs to ensure accuracy, completeness, and internal consistency are automated. Data can be readily downloaded and converted to the format of commercially available statistical software. During collection of the online portion of the study, all data will be transmitted securely using SSL (TLS) 128 bit encryption across the Internet (HTTP). SSL providers users with the assurance of access to a valid, “non-spoofed” site, and prevents data interception or tampering with sensitive information. The SSL certificate that will be used for this project will use 128-bit encryption, the preferred security level of government and financial institutions. 128-bit encryption offers protection that is virtually unbreakable. For example, if a hacker could crack a standard 40-bit SSL session in a day, it is estimated that it would take well beyond a trillion years to accomplish the same thing against a 128-bit SSL session. A dedicated server, which eliminates security issues involved with shared hosting environments where hundreds of websites and users reside on one shared web server as well as ensuring both physical and network security, will be used to house the data. Data will be located in a secured server at UNC Chapel Hill.

The server will be configured with redundant hard drive array to ensure reliability. Access to the data will be password protected within the server’s firewall. Survey responses will be kept separately from participants’ email addresses; the two files will be linked with a non-descript, unique, randomly generally identifier. Only the PI and a designated senior staff member will have the password to access to the “key” that links the nondescript identifier to personally identifiable information. Cookies will not be used in any way to track participant activity.

**What if you want to stop before your part in the study is complete? If at any point in the study you do not want to answer a question or no longer want to participate,** you can stop and withdraw from this studywithout penalty. The investigators also have the right to stop your participation if you have an unexpected reaction, have failed to follow instructions, etc.

**Will you receive anything for being in this study? Will it cost anything? Participants who are asked to watch a one-minute video will have the opportunity to earn up to 150 RMB credit on their mobile phone – this credit will be distributed as twoseparate 100 and 50RMB mobile phone recharges. Participants will receive a 100RMB phone recharge upon completion of the firstquestionnaireand 3-week follow up survey, and 50 RMB for the 3-month follow up survey if that they are eligible for.** There are no costs associated with participating in this research study.

**What if you have questions about this study?** If you have any questions, complaints, or concerns about the research or your participation in the study, feel free to contact Chuncheng Liu at (186-1306-7997).

**What if you have questions about your rights as a research participant?** All research on human volunteers is reviewed by a committee that works to protect your rights and welfare. If you have questions or concerns, or if you would like to obtain information or offer input, please contact the UNC Institutional Review Board at 1-919-966-3113 or by email to [IRB_subjects@unc.edu](mailto:IRB_subjects@unc.edu). You may also contact the Guangdong Provincial Skin Diseases & STI Control Center IRB at 020－83027652 or by email to [sesh@seshglobal.org](mailto:sesh@seshglobal.org).

If you understand and agree to participate in this research study, please select “Agree” from the options below. We thank you for your participation!

- Agree
- Decline (Skip to End of Survey)

**Survey Access (Q6-7)**

6. How did you find out about our research study?

- Blued’s banner ad
- Weibo banner ad
- Weixin banner ad
- Friend referral
- Friend referred me through QQ
- Friend referred me through SMS

7. What device are you using to access our research study?

- Desktop or laptop computer
- Mobile phone
- Tablet device

**A.Sociodemographics (Q8-15)**

*The next set of questions will ask you to provide some information about yourself.*

A6. What province or province-level city do you currently live in?

- Beijing
- Tianjin
- Hebei
- Shanxi
- Inner Mongolia
- Liaoning
- Jilin
- Heilong Jiang
- Shanghai
- Jiangsu
- Zhejiang
- Anhui
- Fujian
- Jiangxi
- Shandong
- Henan
- Hubei
- Hunan
- Guangdong
- Guangxi
- Hainan
- Chongqing
- Sichuan
- Guizhou
- Yun An
- Xizang (Tibet)
- Shaanxi
- Gansu
- Qinghai
- Ningxia
- Xinjiang
- Hong Kong
- Aomen

A7. What city do you currently live in? _____________ (*Text input)*(Do not display if answered 北京，上海，重庆，天津，香港，澳门 to A6）

A8. What is your current legal marital status (referring to women)?

- Not married
- Engaged or Married
- Separated or Divorced
- Widowed

A9. Are you currently enrolled as either a full-time or part-time student?

- Yes
- No

A10. What is the highest level of education that you have**completed**?

- High school or below (including Zhongzhuan)
- Some college (Dazhuan)
- College/Bachelors
- Masters
- PhD

A11. What is your total individual**monthly** income from all sources?

- Less than 1500 RMB
- Between 1500 and 3000 RMB
- Between 3001 and5000 RMB
- Between 5001 and 8000 RMB
- Greater than 8000 RMB

A12. What do you primarily consider yourself to be?

- Gay
- Bisexual
- Straight/Heterosexual
- Transgender
- Unsure/Other

A13. Have you spoken with a physician or other health professional (e.g. HIV testing counselor, pharmacist) about your sexuality or sexual history with men?

- Yes
- No

**B. MSM Basic Situation (Q16-38)**

*The next set of questions will ask you about your sexual behaviors with other men.*

*A “primarypartner” is someone who you havesex with regularly and/or have an emotional commitment to. A “casual partner”is someonewho you havesex withand do not have an emotional commitment to.*

B1. How old were you during your first insertivesexual encounter?

________years old *(Number input)*

B2. Was your firstinsertive sexual encounter with a male or female?

- Male (Skip toB4)
- Female
- Other

B3. How old were you when you had sex with another man for the first time?

________years old *(Number input)*

B4. Were you insertive (1) or receptive (0) during your first sexual encounter with another man?

- Insertive (1)
- Receptive (0)
- Both insertive (1) and receptive (0)

B5. Did you use a condom during your first sexual encounter with another man?

- Yes
- No

B6. In general, where do you usually go to meet your sex partners (Select all that apply)?

- Pub, disco, tearoom, or club
- Spa or bath house, sauna, foot or body massage parlor
- Park, public restroom, public lawn
- Internet
- Other

B7. In the last three months, approximately how many male sex partners have you had?

_________male sex partners *(Number input)*

B8. Of the men you have had sex with in the last three months, would you consider one of them to be aprimary sex partner?

- Yes
- No (Skip to B16)

B9. In the last three months, approximately how many times per week did you have analsex with your primary partner?

_________sex encounters per week

B10. How long have you and your primary sex partner been in a relationship?

- Less than three months
- Between three and six months
- Between six and twelve months
- Between one and two years
- More than two years

B11. In the last three months, when you had anal sex with your primary partner, what role did you assume?

- Always insertive (always 1)(Do not display B15)
- Mostly insertive (mostly 1)
- Both insertive and receptive in similar amounts (Both 1 and 0 in similar amounts)
- Mostly receptive (mostly 0)
- Always receptive (always 0) (Do not display B14)
- No anal sex, only oral sex (Neither 1 nor 0) (Do not display B14 and B15)

B12. In the last three months, when you had sex with your primary partner, how frequently did you or your partner use condoms?

- Never used (Skip to B14)
- Sometimes used
- Mostly used
- Always used (Do not display B14, B15)

B13. In the last three months, when you had sex with your primary partnerdid a condom ever slip off, tear, or otherwise fail?

- Yes
- No

B14.When you are insertive, the reason(s) you do not use a condom with your primary partner include (select all that apply):

- I do not want to use one (e.g. personal preference, uncomfortable)
- Neither of us has a condom
- My partner does not want me to use one
- The condom is of poor quality
- I do not have time to use one
- I believe that my partner is loyal to me
- I am loyal to my partner
- I am drunk or high
- I am HIV negative or I do not believe I am infected with HIV
- My partner is HIV negative or I do not believe he is infected with HIV
- Other

B15.When you are receptive, the reason(s) your primary partner does not use a condom with you include (select all that apply):

- He does not want to use one (e.g. personal preference, uncomfortable)
- Neither of us has a condom
- I do not want him to use one
- The condom is of poor quality
- He does not have time to use one
- I believe that my partner is loyal to me
- He believes that I am loyal to him
- He is drunk or high
- He is HIV negative or does not believe he is infected with HIV
- I am HIV negative or does not believe I am infected with HIV
- Other

B16. In the last three months, have you had sex with another man who was not your primary partner?

- Yes
- No (Skip to B23, Should not say “No” to B8 and B16)

B17. In the last three months, approximately how many times per week did you have analsex (all casual sex partners combined)?

_________sex encounters per week

B18. In the last three months, when you had anal sex with a casual partner, what role did you assume?

- Always insertive (always 1)(Do not display B22)
- Mostly insertive (mostly 1)
- Both insertive and receptive in similar amounts (Both 1 and 0 in similar amounts)
- Mostly receptive (mostly 0)
- Always receptive (always 0) (Do not display B21)
- No anal sex, only oral sex (Neither 1 nor 0) (Do not display B21 and B22)

B19. In the last three months, when you had sex with acasual partner, how frequently did you or your partner use condoms? （Do not display if B17 is “0” or B18 is “No anal sex, only oral sex (Neither 1 nor 0)”)

- Never used (Skip to B21)
- Sometimes used
- Mostly used
- Always used (Do not display B21, B22)

B20. In the last three months, when you had sex with a casual partner did a condom ever slip off, tear, or otherwise fail?

- Yes
- No

B21.When you are insertive, the reason(s) you do not use a condom with a casual partner include (select all that apply):

- I do not want to use one (e.g. personal preference, uncomfortable)
- Neither of us has a condom
- My partner does not want me to use one
- The condom is of poor quality
- I do not have time to use one
- I am drunk or high
- I am HIV negative or I do not believe I am infected with HIV
- My partner is HIV negative or I do not believe he is infected with HIV
- Other

B22.When you are receptive, the reason(s) your casual partner does not use a condom with you include (select all that apply):

- He does not want to use one (e.g. personal preference, uncomfortable)
- Neither of us has a condom
- I do not want him to use one
- The condom is of poor quality
- He does not have time to use one
- He is drunk or high
- He is HIV negative or he does not believe he is infected with HIV
- I am HIV negative or he does not believe I am infected with HIV
- Other

B23. In the last month, did you have any anal sex without a condom with any male partner?(Do not display if answer “1” to B7 and “Always” to B19)

- Yes
- No

**C. Heterosexual Sex Situation (Q39-54)**

*The next set of questions will ask about your sexual behaviors with women.*

*A “primary female partner” is someone who you have sex with regularly, have an emotional commitment to, and/or have married or engaged to be married. A “casual female partner” is someone who you have had sex with but do not have an emotional commitment to.*

C1. Have you ever had vaginal, anal, and/or oral sex with a female partner?

- Yes
- No (Skip to End of Section)

C2. In the last sixmonths, did you have any vaginal and/or anal sex with a female partner?

- Yes
- No (Skip to End of Section)

C3. In the last six months, approximately how many female sex partners have you had?

_________female sex partners *(Number input)*

C4. In the last six months, have you been in a primary relationship with a female partner?

- Yes
- No (Skip to C10)

C5. In the last six months, did you have a concurrent male sex partner outside of your primary relationship with a woman?

- Yes
- No (Do not display C10)

C6. In the last six months, approximately how many times per week did you have vaginal and/or anal sex with your primary female partner?

_________sex encounters per week

C7. In the last six months, when you had sex with your primary female partner, how frequently did you or your partner use condoms?

- Never used (Skip to C9)
- Sometimes used
- Mostly used
- Always used (Do not display C9)

C8. In the last six months, when you had sex with your primary female partner did a condom ever slip off, tear, or otherwise fail?

- Yes
- No

C9. The reason(s) you do not use a condom with your primary female partner include (select all that apply):

- I do not want to use one (e.g. personal preference, uncomfortable)
- Neither of us has a condom
- My partner does not want me to use one
- The condom is of poor quality
- I do not have time to use one
- I believe that my partner is loyal to me
- I am loyal to my partner
- I am drunk or high
- I am HIV negative or I do not believe I am infected with HIV
- My partner is HIV negative or I do not believe she is infected with HIV
- Other

C10. In the last six months, have you had sex with another woman who was not your primary partner?

- Yes
- No (Skip to End of Section if “Always” to C7; otherwise Skip to C15 – Should not answer “No” to C4 and C10)

C11. In the last six months, approximately how many times per week did you have vaginaland/or anal sex (all casual sex partners combined)?

_________sex encounters per week

C12. In the last six months, when you had sex with a casual female partner, how frequently did you or your partner use condoms?

- Never used (Skip to C14)
- Sometimes used
- Mostly used
- Always used (Do not display C14; Skip to End of Section if “Always” to C7)

C13. In the last six months, when you had sex with acasual female partner did a condom ever slip off, tear, or otherwise fail?

- Yes
- No

C14. The reason(s) you do not use a condom with a casual female partner include (select all that apply):

- I do not want to use one (e.g. personal preference, uncomfortable)
- Neither of us has a condom
- My partner does not want me to use one
- The condom is of poor quality
- I do not have time to use one
- I am drunk or high
- I am HIV negative or I do not believe I am infected with HIV
- My partner is HIV negative or I do not believe she is infected with HIV
- Other

C15. In the last month, did you have sex without a condom with any female partner?(Do not display if answer “1” to B7 and “Always” to B19)

- Yes
- No

**D. Sexual Behavior (Q55-63)**

*The next set of questions will ask about any “risky” sexual behaviors that you may or may not have engaged in with other men and/or women.*

D1. In the last three months, did you ever have sex while you were drunk (from drinking alcohol)?

- Yes
- No

D2. In the last three months, was you partner ever drunk (from drinking alcohol) while you had sex?

- Yes
- No (Skip to D4 if “No” for D1 and D2)

D3. In the last three months, how often did you have sex while you and/or your partner was drunk?

- Never
- Rarely
- Occasionally/Sometimes
- Very often
- Always

D4. In the last twelve months, did you ever use “meth” before or during sex?

- Yes
- No

D5. In the last twelve months, did you ever participateingroup sex with other men?

- Yes (Display D6)
- No

D6.During your most recent group sex experience, did you have any anal sex without a condom?

- Yes
- No

D7. In the last twelve months, were you ever paid (with money or gifts) to have sex?

- Yes
- No (Skip to End of Section)

D8. In the last twelve months, has your main source of income come from having sex with customers?

- Yes
- No

D9. In the last twelve months, have you ever paid (with money or gifts) a man to have sex?

- Yes
- No

**E. Sex Tourism (Q64-79)**

*The next set of questions will ask about leaving your city and/or China to purchase sex.*

E1. Have you ever purchased sex (with money or gifts) while traveling outside of your city of residence?

- Yes
- No (If “No” skip to E22)

E2. Have you ever traveled outside of your city of residence with the primary purpose of purchasing sex?

- Yes
- No

E3. When you traveled to purchase sex, did you travel within China or leave the country?

- Within China (Display E4a)
- Outside China (Display E4b)
- Both (Display E4a and E4b)

E4a. Which city/cities in China did you travel to when you purchased sex? _________ *(Text Input*)

E4b. Which country/countries and cities did you travel to when you purchased sex? _____ (*Text Input*)

E5. How did you arrive at your destination?

- Car
- Train
- Airplane
- Ship

E6. Why did you decide to purchase sex while traveling?

- I was afraid of seeing someone I know in my hometown
- Sex is less expensive at the location I traveled to
- There was less likelihood that I would have to use a condom if I purchase sex
- I am unable to purchase sex in my hometown
- I wanted to try sexual intercourse with another gender
- I was drunk or using drugs, I did not plan it

E7. When you purchased sex while outside your city of residence, who did you purchase sex from (select all that apply)?

- Men
- Women
- Transgender

E8a. When you purchased sex while outside your city of residence, have you ever had any vaginal sex without a condom? (Display if “Women” or “TG” for E7)

- Yes (Display E17)
- No

E8b. When you purchased sex while outside your city of residence, have you ever had any anal sex without a condom?

- Yes (Display E17)
- No

E9. Once you were at your travel destination (during your most recent trip abroad), how did you find someone to purchase sex from (select all that apply)?

- Mobile app portal
- Online (not an app) portal
- In-person proposition
- Local establishment

E10. During your most recent experience when you purchased sex while abroad, approximately how many sex partners did you purchase? (Please enter “0” partners if no partners of the following type)

_________malesex partners *(Number input)*

_________femalesex partners*(Number input)*

_________transgendersex partners*(Number input)*

E11. During your most recent experience when you purchased sex while traveling, approximately how much did you pay (RMB) for your last sex encounter?

_________ *(Text Input*)

E12. During your most recent experience when you purchased sex while traveling, of what nationality was your last partner?

_________ *(Text Input*)

E13. During your most recent experience when you purchased sex while traveling, the reason(s) ­­­­you did not use a condom include (select all that apply):

- I did not want to use one (e.g. personal preference, uncomfortable)
- I did not want my partner to use one
- Neither of us had a condom
- My partner did not want to use one (e.g. personal preference, uncomfortable)
- My partner did not want me to use one
- The condom was of poor quality
- I did not have time to use one
- My partner did not have time to use one
- I was drunk or high
- My partner was drunk or high
- I am HIV negative or I do not believe I am infected with HIV
- My partner was HIV negative or I do not believe my partner was infected with HIV

E14. How strongly do you agree with the following statement: During my most recent experience purchasing sex while traveling, I behaved with less caution than I normally would while at home

- Strongly yes
- Yes
- The same
- No
- Strongly No

E15. Did you travel alone or with others?

- Alone
- With others

E16. During your most recent experience when you purchased sex while traveling, did you ask your partner about his/her HIV status before having sex?

- Yes
- No

**F. Condom Behavior(Q80-96)**

*The next set of questions will ask about your practices and attitudes in regards to condom use.*

F1. In the last three months, how often did you carry a condom with you when there was the possibility you may have sex later?

- Always
- Sometimes
- Hardly ever
- Never

F2. If you needed a condom, where is the first place you would go to find one?

- Pharmacy or drugstore
- Supermarket
- Health clinic
- Community event
- Restroom vending machine
- Friend
- Partner
- Other

F3. If I had sex and told my friends that I did not use a condom, they would be angry or disappointed.

- Strongly agree
- Agree
- Neutral
- Disagree
- Strongly disagree

F4. My friends talk a lot about “safer" sex.

- Strongly agree
- Agree
- Neutral
- Disagree
- Strongly disagree

F5. My friends and I encourage each other before dates to practice "safer" sex.

- Strongly agree
- Agree
- Neutral
- Disagree
- Strongly disagree

F6. If I thought that one of my friends had sex on a date, I would ask them if they used a condom.

- Strongly agree
- Agree
- Neutral
- Disagree
- Strongly disagree

F7. If a friend knew that I might have sex on a date, he/she would ask me if I was carrying a condom.

- Strongly agree
- Agree
- Neutral
- Disagree
- Strongly disagree

F8. When I think that one of my friends might have sex on a date, I would ask him/her if he/she was carrying a condom.

- Strongly agree
- Agree
- Neutral
- Disagree
- Strongly disagree

F9. If I might have sex on a date and I do not have a condom, I would make an effort to go out of my way and get one.

- Strongly agree
- Agree
- Neutral
- Disagree
- Strongly disagree

F10. I would feel comfortable discussing condom use with a potential partner before we engaged in sex.

- Strongly agree
- Agree
- Neutral
- Disagree
- Strongly disagree

F11. I would feel comfortable letting a primary partner know that I want to have sex with a condom.

- Strongly agree
- Agree
- Neutral
- Disagree
- Strongly disagree

F12. I would feel comfortable letting a casual partner know that I want to have sex with a condom.

- Strongly agree
- Agree
- Neutral
- Disagree
- Strongly disagree

F13. I feel confident that I could refuse to have sex with a partner who did not want you to use a condom.

- Strongly agree
- Agree
- Neutral
- Disagree
- Strongly disagree

F14. I feel confident in my ability to incorporate putting a condom on myself or my partner into foreplay.

- Strongly agree
- Agree
- Neutral
- Disagree
- Strongly disagree

F15. I feel confident that I could use a condom with a partner without "breaking the mood."

- Strongly agree
- Agree
- Neutral
- Disagree
- Strongly disagree

F16. In the last three months, did you ever **try** to convince a partner who did not want to use a condom to use one before having sex?

- Yes, and I was successful
- Yes, but I was unsuccessful
- No

F17. In the last three months, did your partner every **try** to convince you to use a condom when you did not want to use one before having sex?

- Yes, and he was successful
- Yes, but he was unsuccessful
- No

**G. HIV/STI Testing (Q97-132)**

*The next set of questions will ask about your HIV and STI testing and results.Self-testing refers to you administering the test yourself and interpreting results.*

G1. Have you ever been tested for HIV?

- Yes
- No (Skip to G25)

G2. Have you ever given or received an HIV self-test?

- Yes
- No

G3. Have you ever self-tested for HIV?

- Yes
- No (Skip to G21)

G4. Did someone else force you to take an HIV self-test?

- Yes
- No

G5. Who was with you when you self-tested? (Can select multiple)

- No one, I was alone
- Partner
- Friend

G6. Was your HIV self-test the first time you ever tested for HIV?

- Yes (Skip to G8)
- No

G7. What happened to your HIV testing frequency after you first used a self-test?

- Increased
- Decreased
- No change

G8. Have you ever received a positive result with HIV self-testing?

- Yes (Show G15-G16)
- No (Skip to G11)

G9.Has using an HIV self-test caused you subsequent suicidal feelings?

- Yes
- No

G10.Has using an HIV self-test led to a violent confrontation (physically hitting)?

- Yes
- No

*The next set of 4 questions will ask you to recall experiences specific to self-testing.*

G11. Has using an HIV self-test has increased your desire to seek follow-up care, as opposed to other forms of HIV testing?

- Yes
- No

G12.Self-testing for HIV gives me a sense of empowerment by allowing me to choose when I test.

- Strongly Agree
- Agree
- Neutral
- Disagree
- Strongly Disagree

G13.Self-testing for HIV gives me a sense of empowerment by allowing me to choose where I test.

- Strongly Agree
- Agree
- Neutral
- Disagree
- Strongly Disagree

G14. Self-testing for HIV gives me a sense of empowerment by allowing me to choose with whom I test.

- Strongly Agree
- Agree
- Neutral
- Disagree
- Strongly Disagree

G15. Did you confirm your positive HIV self-test result at the CDC or hospital?

- Yes
- No

G16. Did you receive post-test counseling?

- Yes (show G17)
- No

G17. What kind of post-test counseling did you receive?

- online
- telephone
- in-person

G18. Where did you obtain your HIV self-test kit?

- online
- hospital
- pharmacy
- CBO
- friend

G19. Was your HIV self-test oral or blood?

- Oral
- Blood

G20. In the last two years, how frequently did you get tested for HIV?

- Less than once every two years
- Once a year
- Once every six months
- Once every three months
- Monthly

G21. What was the result of your most recent HIV test?

- HIV positive/infected (Display G23)
- HIV negative/uninfected
- I never got my test results

G22. Did you notify your primary male sex partner about your most recent HIV test result?

- Yes
- No
- I do not have a regular partner (Do not display G25)

G23. Have you ever taken anti-retroviral therapy (ART) for your HIV infection?

- Yes – I have taken, and I am currently taking
- Yes – I have taken, but I am currently not taking (Display G24)
- No – I have never taken

G24. Why did you stop taking ART? (Select all that apply)

- It was too expensive
- I didn’t like the side effects
- I didn’t feel that it was working
- I thought it was cumbersome (too much time, forgot to take, etc.)
- Stigma

G25. Has your primary male sex partner ever been tested for HIV? (Do not display if yes to C4 or yes to B8)

- Yes
- No (Skip to G27)

G26. What was the result of your primary male sex partner’s most recent HIV test?

- HIV positive/infected
- HIV negative/uninfected
- Never got test results
- I don’t know

G27. Have you ever had a male sex partner who tested HIV positive?

- Yes
- No (Skip to G30)
- I don’t know (Skip to G30)

G28. Did you ever have any anal sex without a condom with a HIV positive partner?

- Yes
- No

G29. Approximately how many HIV positive male sex partners have you had?

_________sex partners *(Number input)*

G30. Have you ever been tested for syphilis?

- Yes
- No (Skip to G36)

G31. Have you ever used a self-testing kit for syphilis?

- Yes
- No (Skip to G36)

G32. Was your self-test the first time you ever tested for syphilis?

- Yes (Skip to G34)
- No

G33. What happened to your syphilis testing frequency after you first used a self-test?

- Increased
- Decreased
- No change

G34. Where did you obtain your syphilis self-test kit?

- online
- hospital
- pharmacy
- CBO
- Friend

G35. Have you ever performed syphilis and HIV self-testing together?

- Yes
- No

G36. In the last twelve months, which of the following services did you receive (Select all that apply):

- Condom distribution
- Lubricant distribution
- Peer Education
- STD Diagnosis or Treatment
- HIV counseling or Testing
- AIDS/STD Materials (pamphlets, etc.)

**I. Community Engagement (Q133-143)**

*The next set of questions will ask you about your experiences with activities in your community promoting sexual health.*

I1. In the last three weeks, have you viewed any videos promoting condom useamong MSM?

- Yes
- No

I2. In the last three weeks, have you viewed any videos promoting HIV testingamong MSM?

- Yes
- No

I3. Are you aware of any ongoing community events promoting sexual health among MSM?

- Yes
- No

I4. Have you ever helped organize a testing and/or awareness campaign (e.g. HIV, condom use, etc.) that promoted sexual healthamong MSM?

- Yes
- No

I5. Have you ever volunteered at a health clinic or other location that provided sexual health servicesamong MSM?

- Yes
- No

I6. Have you ever encouraged someone else to get tested for HIV and/or another sexually transmitted disease?

- Yes
- No

I7. Have you ever accompanied a friend or partner to a testing facility to get tested for HIV and/or another sexually transmitted disease?

- Yes
- No

I8. How important to you is community engagement and participation in developing sexual health campaigns (for your own community)?

- Very important
- Important
- Neither important or not important
- Slightly important
- Not important

I9. Have you ever participated in online forums or discussions on social media (ie.Weixin, Weibo, Twitter, or other on-line communities) about about sexual health, condom use, or HIV/STD testing or related services?

- Yes
- No

I10. Do you have a Weibo account?

- Yes (Display I11)
- No

I11. How many Weibo followers do you have?

- Less than 100
- 101-500
- 501-1000
- 1001-1500
- 1501-2000
- More than 2001

**Follow-up Contact (Q144-145)**

FUC1. Thank you for taking the time to complete our survey! Based on your responses to our questionnaire, we request that you to complete a follow-up survey in three weeks’ time. Upon completion of this survey, you will receive an additional 50 RMB mobile phone recharge! When the time comes, we would like to send you a reminder to complete the survey via QQ. Will you agree to provide us your QQ number? If you agree,you will be contacted by the following user:

Number: 2663701478

Name: 赛思研究团队

- Agree (Display FUC2)
- Disagree

FUC2. Please input your QQ number:

- QQ number:_________

**Referral (Q146)**

R1. If you think any of your male friends would be interested in participating in our research survey, please share our study with them! Alternatively, you can provide us with either their mobile phone or QQ number, and we will send them a link to our survey. (Please enter as many unique numbers as you are willing in the spaces provided.)

If you provide a QQ number for referral, please notify your friend(s) that they will be contacted by赛思研究团队 (#: 2663701478).

If you provide a mobile phone number for referral, please notify your friend(s) that they will be contacted by 18613067997.

- Mobile Phone #s:_________
- QQ numbers:_________

**End of Survey**

**“Survey Video” (Crowdsourced and Social Marketing)**

We would now like you to watch a short-one minute video.Please make sure to view the entire video before closing the window.The video may take up to a minute to load, thank you for your patience.

**“Mobile Confirmation”**

Please confirm your mobile phone number at this time to receive our reminder of the follow-up survey and reward. Please notice that only after you finish the 3 week follow up could you get the 100 top up reward.

**“3 Weeks Follow-upSurvey**”

Thank you again for completingour survey threeweeks ago. If you complete the following survey you will receive a 100RMBrecharge on your mobile phone.(SESH Project)

**“Reward - Mobile TopUp”**

Thank you very much for taking part in our survey. Your efforts are greatly appreciated and will contribute towards improving sexual health of MSM across China.

**4 Weeks Follow-up(To participants whodid not respond at 3 weeks)**

Thank you again for completingour survey 4 weeks ago. If you complete the following survey you will receive a 100 RMB recharge on your mobile phone.

**“3 Months Follow-upSurvey”**

Thank you again for completingour survey 3monthsago. If you complete the following survey you will receive a 50 RMB recharge on your mobile phone.

**“Reward - Mobile TopUp”**

Thank you very much for taking part in our survey. Your efforts are greatly appreciated and will contribute towards improving sexual health of MSM across China.

**3Months + 1 WeekFollow-up(To participants whodid not respond at 3 months)**

Thank you again for completing our survey 3monthsago. If you complete the following survey you will receive a 50 RMB recharge on your mobile phone.
